# *Lactiplantibacillus argentoratensis* AGMB00912 alleviates salmonellosis and modulates gut microbiota in weaned piglets: a pilot study

**DOI:** 10.1038/s41598-024-66092-z

**Published:** 2024-07-05

**Authors:** Ki-Nam Yoon, Han Gyu Lee, Seo-Joon Yeom, Sang-Su Kim, Jong-Heum Park, Beom-Seok Song, Seung-Won Yi, Yoon Jung Do, Byungkwan Oh, Sang-Ik Oh, Jong-Bang Eun, Seung-Hwan Park, Ju Huck Lee, Hyeun Bum Kim, Ju-Hoon Lee, Tai-Young Hur, Jae-Kyung Kim

**Affiliations:** 1 Research Division for Biotechnology, Advanced Radiation Technology Institute, Korea Atomic Energy Research Institute, 29 Geumgu-gil, Jeongeup-si, Jeollabuk-do 56212 Republic of Korea; 2 Division of Animal Diseases and Health, National Institute of Animal Science, Rural Development Administration, Wanju-gun, Jeollabuk-do 55365 Republic of Korea; 3 Biosafety Research Institute and College of Veterinary Medicine, Jeonbuk National University, Iksan, Jeollabuk-do 54596 Republic of Korea; 4 Department of Food Science and Technology, Graduate School of Chonnam National University, Gwangju, 61186 Republic of Korea; 5 Korean Collection for Type Cultures, Korea Research Institute of Bioscience and Biotechnology, Jeongeup-si, 56212 Republic of Korea; 6 Department of Animal Resources Science, Dankook University, Cheonan, 31116 Republic of Korea; 7 Department of Food and Animal Biotechnology, Department of Agricultural Biotechnology, Research Institute of Agriculture and Life Sciences, Center for Food and Bioconvergence, Seoul National University, Seoul, 08826 Republic of Korea

**Keywords:** Microbiome, Metagenomics

## Abstract

This study aimed to evaluate the efficacy of *Lactiplantibacillus argentoratensis* AGMB00912 (LA) in reducing *Salmonella* Typhimurium infection in weaned piglets. The investigation focused on the influence of LA on the gut microbiota composition, growth performance, and *Salmonella* fecal shedding. The results indicated that LA supplementation significantly improved average daily gain and reduced the prevalence and severity of diarrhea. Fecal analysis revealed reduced *Salmonella* shedding in the LA-supplemented group. Furthermore, LA notably altered the composition of the gut microbiota, increasing the levels of beneficial Bacillus and decreasing those of harmful Proteobacteria and Spirochaetes. Histopathological examination showed less intestinal damage in LA-treated piglets than in the controls. The study also observed that LA affected metabolic functions related to carbohydrate, amino acid, and fatty acid metabolism, thereby enhancing gut health and resilience against infection. Short-chain fatty acid concentrations in the feces were higher in the LA group, suggesting improved gut microbial activity. LA supplementation enriched the population of beneficial bacteria, including *Streptococcus*, *Clostridium*, and *Bifidobacterium*, while reducing the number of harmful bacteria, such as *Escherichia* and *Campylobacter*. These findings indicate the potential of LA as a probiotic alternative for swine nutrition, offering protective effects to the gut microbiota against *Salmonella* infection.

## Introduction

Preventing salmonellosis caused by *Salmonella enterica* subspecies *enterica* serovar Typhimurium (*S.* Typhimurium*,* ST) is crucial in industrial swine rearing, especially in weaned piglets. During the weaning phase, separation from the mother sow and transition to solid feed can decrease microbial diversity and promote intestinal inflammation^1^. This gut environment can provide physiological advantages to pathogenic microorganisms, such as the production of tetrathionate by the reactive oxygen species generated during intestinal inflammation. Tetrathionate is produced when these oxygen radicals react with thiosulfate, a compound that is derived from the hydrogen sulfide produced by fermentative gut commensals^2,3^. This process, in turn, can facilitate the proliferation of *Salmonella* by providing it with a new electron acceptor to support anaerobic respiration, giving it a competitive advantage over other microorganisms that do not utilize tetrathionate^2,4^. A typical characteristic of salmonellosis in weaned piglets is the high concentration of ST excreted in the feces at an early stage of infection, causing inflammation in the small and large intestines, which in turn leads to post-weaning diarrhea^5–8^. These intestinal disorders accelerate the disruption of microbial diversity and gut homeostasis primarily through infection with pathogenic microorganisms^9,10^. This process can lead to decreased feed intake, negatively affect growth performance, and sometimes result in swine mortality and financial loss to the breeders^11–14^. Therefore, preventing salmonellosis plays a crucial role in the weaning phase, and it may involve promoting gut homeostasis and maintaining piglet health.

Antibiotics such as sulfamethoxazole and trimethoprim have been used as feed additives to suppress salmonellosis in the swine industry; however, their use has been restricted because of the identification of multi-antibiotic-resistant *Salmonella* strains and the occurrence of enteric dysbiosis^15–17^. As an alternative, probiotic strains have been explored as feed additives because of their ability to alleviate intestinal disorders, modulate the immune response, inhibit the proliferation of pathogens, and stabilize the gut microbiota by producing beneficial bioactive compounds, including SCFAs, bacteriocins, enzymes, and vitamins^18,19^. Among these probiotics, *Lactiplantibacillus plantarum* includes the subspecies *L. argentoratensis*, a gram-positive, bacillus-shaped, facultative anaerobic bacterium that was first isolated from cassava, tapioca, and white maize. It is distinguished from *L. plantarum* subsp. plantarum by its unique gene profile and its inability to metabolize certain sugars^20^. *Lactiplantibacillus argentoratensis* is notable for its fermentative abilities, utilizing both homo- and heterofermentation pathways to produce various beneficial compounds, such as lactate, acetate, formate, and SCFAs, which are essential for gut health^21,22^. During acetate production, this pathway could also produce carbon dioxide and hydrogen peroxide. Oxygen undergoes a Mn-dependent process to be converted to hydrogen peroxide. In this process, a catalase reducing the oxygen concentration—a favorable condition for aerotolerant bacteria^23^.

Moreover, *L. plantarum*, including strains like those discussed by Liu et al.^24^, has shown strain-specific properties for the prevention of Salmonella infection, highlighting its potential as a functional feed additive in piglets^21,22,25^. Similarly, Dell’Anno et al.^26^ also illustrate the effectiveness of *L. plantarum* and *L. reuteri* in preventing diarrhea in weaned piglets, further substantiating the beneficial application of these probiotics^25,27^. Specifically, SCFAs are key metabolites in probiotics that play an important role in maintaining gut health by modulating pH in the gastrointestinal tract, promoting epithelial cell proliferation, and acting as mediators at the interface between the host and its microbiome^22,28,29^.

In our previous studies, we isolated *L. argentoratensis* AGMB00912 (LA) from the stool of healthy swine. Through an in vitro evaluation, we revealed that its antimicrobial activity against pathogenic microorganisms was mediated by the production of SCFAs (data not published). However, studies in weaned piglets are essential to confirm the efficacy of LA in promoting gut health and growth performance. Therefore, this in vivo pilot study aimed to test the hypothesis that weaned piglets, with enhanced intestinal stability from dietary supplementation with LA, might present with reduced rates of the ST infection.

## Results

### Growth performance and S. Typhimurium fecal shedding

**Figure 1:**
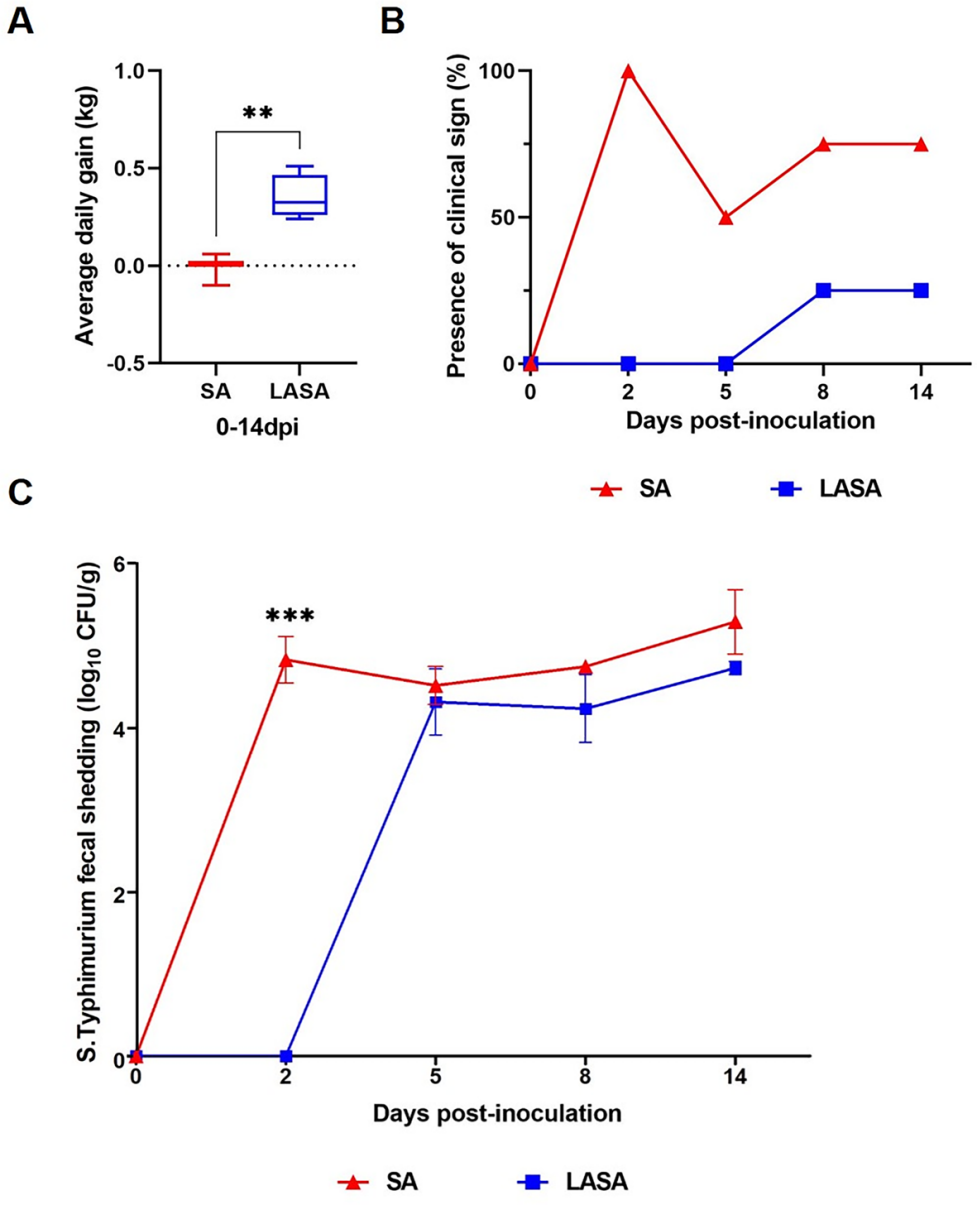
*Salmonella* fecal shedding and clinical data of LASA and SA weaned pigs. (**A**) Average daily gain in LASA and SA group during the experiment. (**B**) Percentage of clinical observation in LASA and SA. (**C**) Total *S*. Typhimurium shedding in fecal samples from LASA and SA at different (0, 2, 5, 8, and 14 dpi) time points.

We compared the ADG of the LASA and SA groups (Fig. 1A). The ADG in the LASA group (0.35 kg/day) was significantly (*P* = 0.006) higher than that in the SA group (− 0.01 kg/day) during the ST infection period. Diarrhea was the most prevalent in the fecal samples of the SA group at 2 dpi (100%), followed by 8 and 14 dpi (75%), and 5 dpi (50%). In contrast, the LASA group exhibited clinical signs only at 8 and 14 dpi (25%), with no observable clinical signs at the other time points (Fig. 1B). The ST fecal shedding level in the SA group increased from 0 to 2 dpi, whereas that in the LASA group showed only a slight increase from 2 to 5 dpi (Fig. 1C). In the early stages of the ST infection (0–2 dpi), fecal shedding was significantly lower (*P* < 0.001) in the LASA group than that in the SA group. After 2 dpi, the LASA group consistently exhibited a tendency toward lower fecal shedding, compared to the SA group, although the difference was not statistically significant. These findings indicated that dietary supplementation with LA may prevent *Salmonella* infection at 2 dpi, and also have tendency to reduce early *Salmonella* infections.

### SCFA concentration

**Figure 2:**
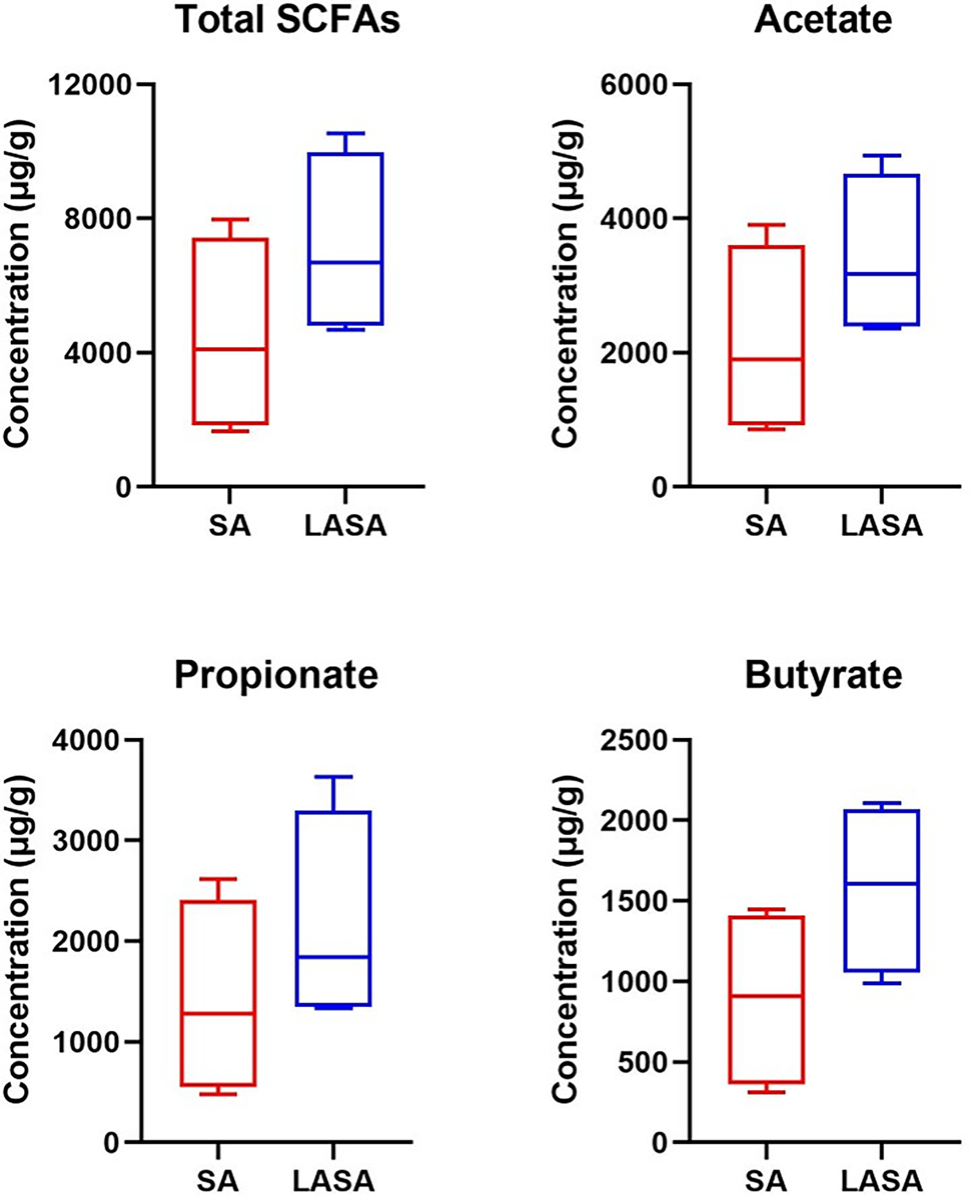
Fecal concentration of three SCFAs (acetate, propionate, and butyrate) in LASA and SA.

At 14 dpi, the total fecal SCFA concentrations were 4452.0 ± 2960.7 and 7147.8 ± 2758.5 μg/g in the SA and LASA groups, respectively (Fig. 2). The fecal concentrations of acetate (2142.0 ± 1432.0 μg/g), propionate (1416.5 ± 981.0 μg/g), and butyrate (893.5 ± 561.7 μg/g) in SA piglets were lower than those in LASA piglets (acetate: 3409.8 ± 1223.0, propionate: 2162.3 ± 1073.2, butyrate: 1575.8 ± 542.5 μg/g). However, no significant differences were observed in the total SCFAs, acetate, butyrate, and propionate concentrations between the LASA and SA groups.

### Histopathological lesions

**Figure 3:**
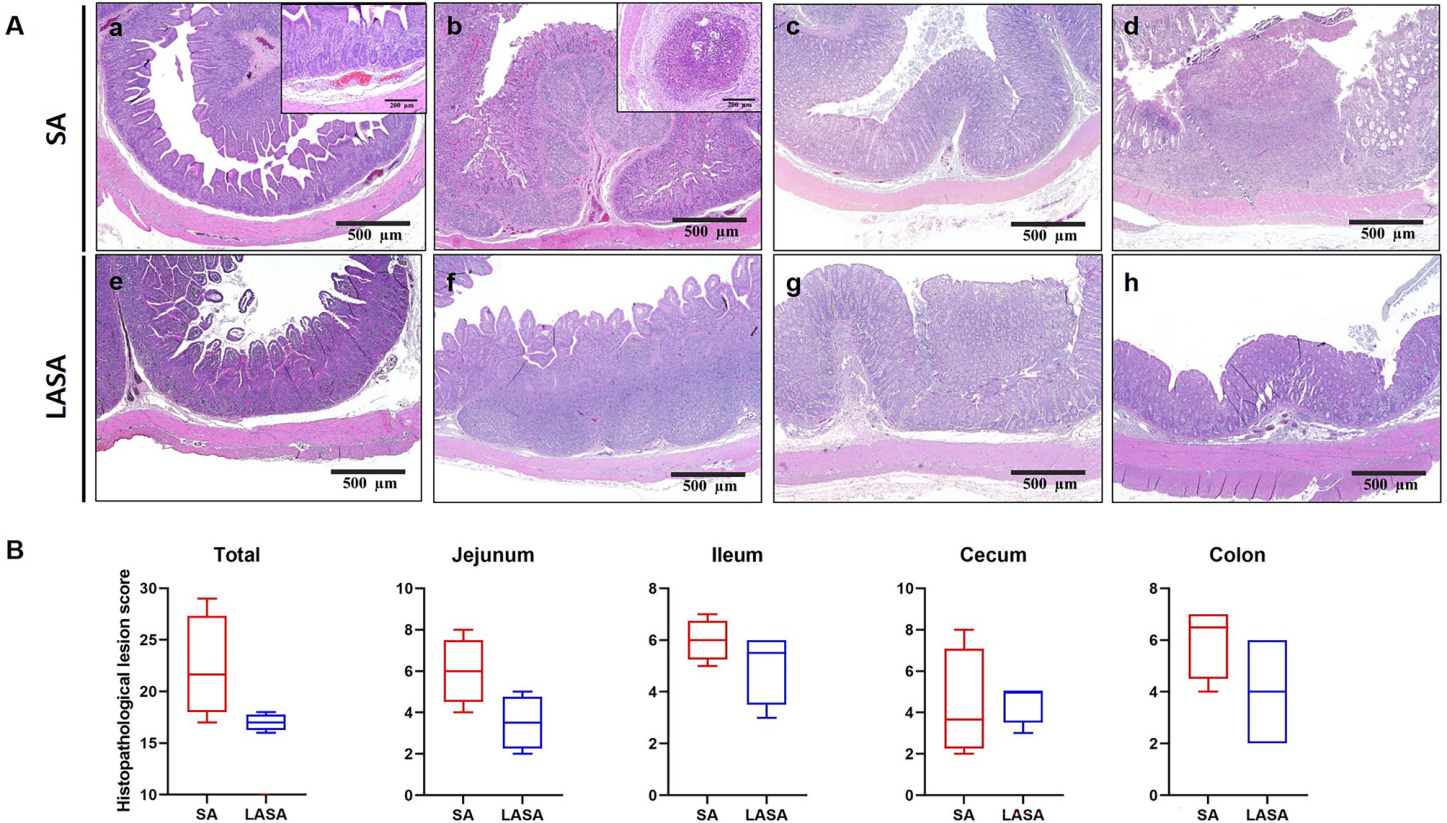
Histopathological lesion in weaned pigs. (**A**) Representative hematoxylin and eosin staining of the intestinal tissues from experimental pigs. (a) Jejunum from SA group. Villous erosion and congestion in submucosa (inset). (b) Ileum from SA group. Congestion in villus crypt and crypt abscess (inset). (c) Cecum from SA group. (d) Colon from SA group. Ulcerative colitis. (e) Jejunum from LASA group. Mild congestion in villus crypt. (f) Ileum from LASA group. (g) Cecum from LASA group. (h) Colon from LASA group. (**B**) Histopathological scores of the intestine (jejunum, ileum, cecum, and colon) samples.

The histopathological scores of the four intestinal segments (jejunum, ileum, cecum, and colon) were measured to evaluate the effect of LA supplementation on the intestine. Although histopathological lesions were observed in both groups (Fig. 3A), the SA group exhibited more severe intestinal damage than did the LASA group, as indicated by the total average histopathological lesion scores in the jejunum, ileum, cecum, and colon (SA, 22.33; LASA, 17.00) (Fig. 3B). Among the assessed parameters, ulcerations and crypt abscesses were more severe in the SA group than in the LASA group which is along with diarrhea result. Furthermore, the average scores for each intestinal region for the SA and LASA groups were as follows: jejunum, 6.0 and to 3.5; ileum, 6.0 and 5.0; cecum 4.3 and 4.5, and colon 6.0 and 4.0, respectively.

### LA supplementation modulates the gut microbiota composition in a ST-infected piglet

**Figure 4:**
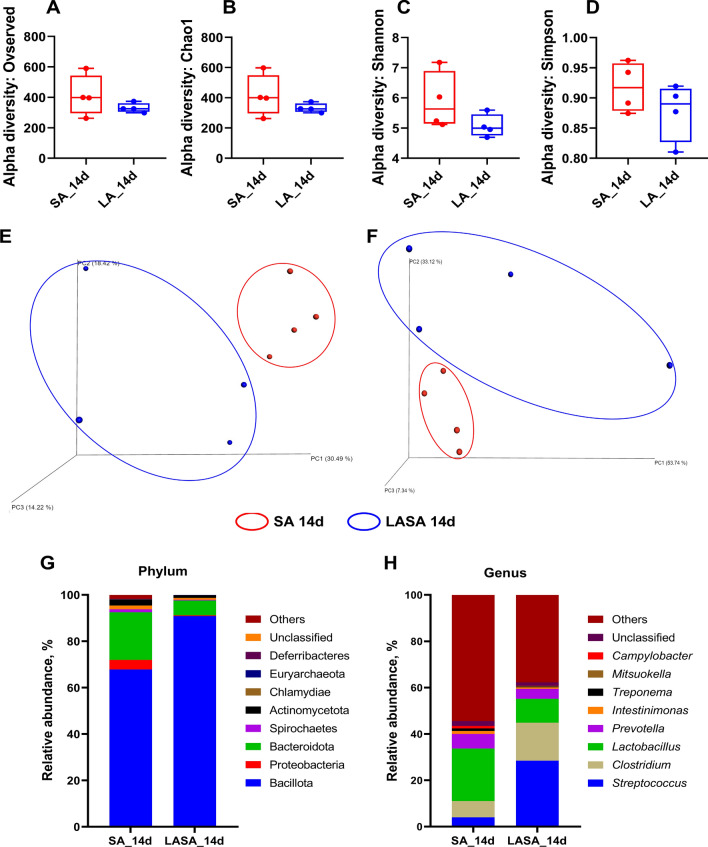
Box plots of the alpha diversity indices in the gut microbiomes of weaned piglets treated with SA and LASA. Species richness levels were measured using (**A**) observed features and (**B**) Chao1 diversity indices. Species evenness markers were measured using (**C**) Shannon and (**D**) Simpson diversity indices. Principal coordinate analysis plots of different groups of weaned piglets revealed that the SA (red oval) and LASA (blue oval) groups were significantly clustered based on unweighted (**E**) and weighted (**F**) UniFrac distance metrics. Gut microbiota composition at the phylum and genus level with SA and LP treatment. Bar plots show the relative abundance of each mouse group taxa at the phylum (**G**) and genus H) level.

To investigate the effects of LA on intestinal microbiota diversity in weaned piglets subjected to ST infection, we analyzed the V3-V4 region of the 16S rRNA gene amplicon sequencing in stool samples collected at 14 days post-infection. Additional file 1 displays the alpha rarefaction curves obtained from 16S rRNA gene sequencing, which delineate species richness in the fecal samples from both the SA and LASA groups. The curves were plotted following the normalization of sequence counts to the minimum observed between the groups, ensuring comparability and accounting for variations in sequencing depth. The alpha-diversity index (observed features, Chao1, Shannon, and Simpson) in the fecal samples of weaned piglets is shown in Fig. 4A–D. The SA and LASA groups exhibited no significant differences in the observed features or Chao1, Shannon, or Simpson indices (*P* > 0.05). Moreover, we performed community structure analysis (beta diversity) using PCoA. PCoA plots based on both unweighted (Fig. 4E) and weighted UniFrac (Fig. 4F) distances showed significant differences in the separation of microbial communities between the SA and LASA groups of weaned piglets (*P* = 0.023 and 0.027, respectively). These results indicate that dietary supplementation with LA modulates the gut microbiota, with significant differences observed in the bacterial communities of weaned piglets between the SA and LASA groups.

We examined the proportions of the bacterial communities in weaned piglets infected with ST (Fig. 4G–H). At the phylum level, the SA group showed a relative abundance of 67.84% in Bacillota, whereas Proteobacteria and Spirochaetes accounted for 4.12% and 1.26% of the species, respectively. In contrast, the LASA group exhibited the Bacillota proportion of 90.82%, and Proteobacteria and Spirochaetes proportions of 0.33% and 0.27%, respectively (Fig. 4G). These results suggest that LA treatment may prevent ST-induced increases in Proteobacteria and Spirochaetes levels, while promoting an increase in Bacillota levels. At the genus level, *Streptococcus* and *Clostridium* were the predominant genera in the LASA group, constituting 28.49% and 16.36% of the sample, respectively. In contrast, these genera were significantly less abundant in the SA group, with *Streptococcus* at 4.02% and *Clostridium* at 7.03%. In particular, the relative abundances of the genera *Campylobacter* and *Treponema* in the SA group were 1.07% and 1.05%, respectively, which decreased with LA supplementation, exhibiting a pattern similar to that observed for Proteobacteria and Spirochaetes at 0.03% and 0.27%, respectively. This finding suggests that *Campylobacter* and *Treponema* may be the main genera contributing to the changes in Proteobacteria and Spirochaetes levels (Fig. 4H).

**Figure 5:**
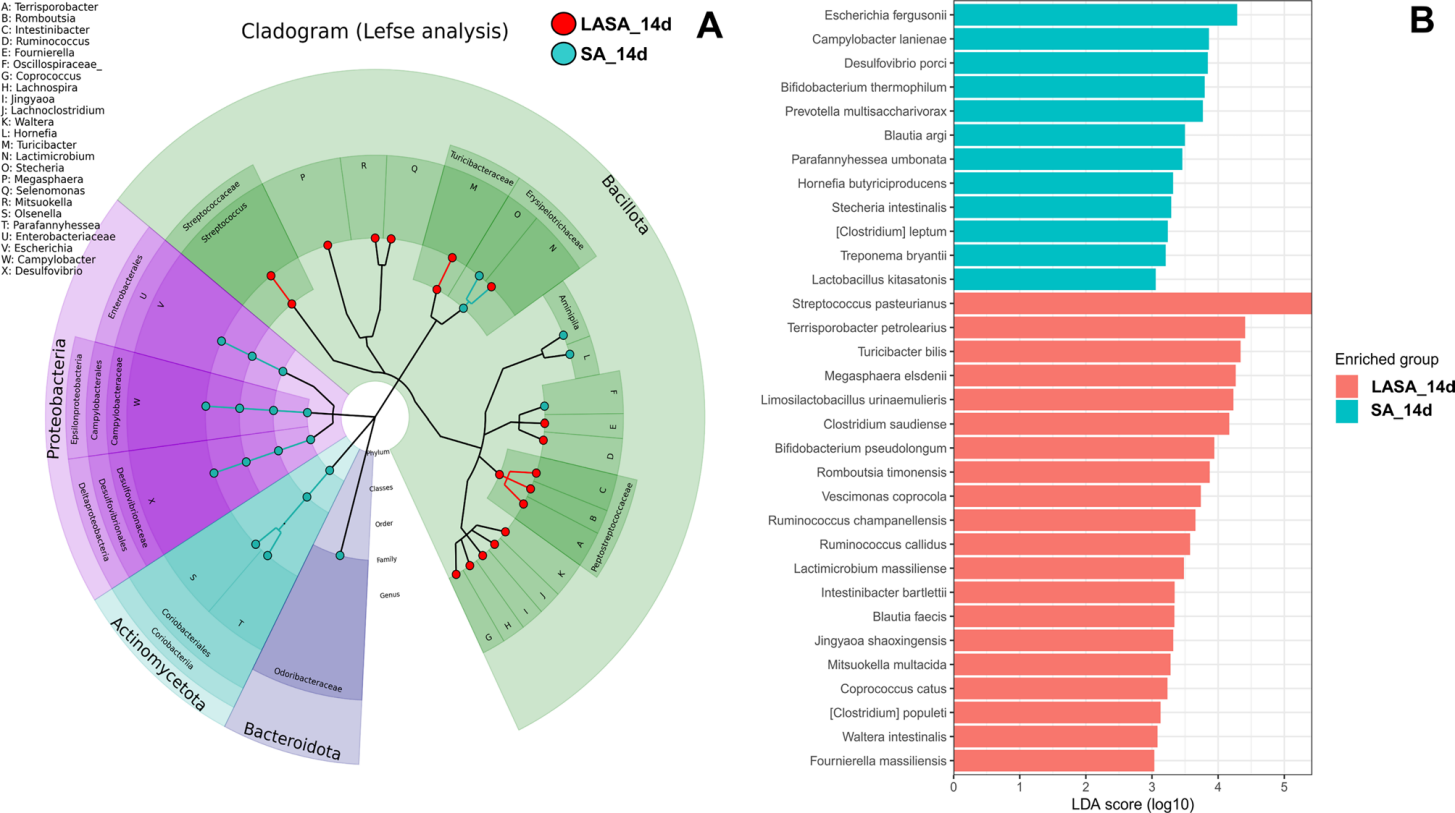
Differential abundance of bacteria among the SA group as determined using the linear discriminant analysis effect size algorithm. *P*-values of < 0.05 were deemed significant in both the Kruskal–Wallis and Wilcoxon tests. The cladogram shows differential abundance at the phylum, class, order, family, and genus levels (**A**). The histogram depicts differential abundance at the species level (**B**). A discriminative feature had a log10 LDA score of 3. The length of each histogram represents the LDA score, indicating the degree of influence of each species.

To examine the differences in gut microbiota taxa between the SA and LASA groups, the LEfSe algorithm was used (Fig. 5A–B). The LEfSe analysis-based cladogram was visualized from the phylum to the genus levels (Fig. 5A), and histograms (Fig. 5B) exhibited significantly different abundances at the species level, as indicated by the LDA score (LDA > 3).

The LEfSe results provides a detailed view of the microbial taxa that are differentially abundant between the SA and LASA groups. Notably, the SA group exhibited a significantly higher abundance of genera such as *Escherichia*, *Campylobacter*, *Desulfovibrio*, and *Treponema*, which are typically associated with pathogenicity and may contribute to dysbiosis within the gut microbiome. Conversely, the LASA group showed a pronounced increase in beneficial or commensal genera including *Streptococcus*, known for its lactic acid production; *Terrisporobacter*, which has been linked to anti-inflammatory properties; and Bifidobacterium, a genus widely recognized for its probiotic potential. Furthermore, the increased abundance of *Clostridium* in the LASA group may suggest an environment that supports the production of SCFAs, conducive to maintaining gut health and integrity. These alterations in microbial abundance paint a picture of the impact that LA supplementation can have on shaping the gut ecosystem, potentially modulating it towards a configuration that supports the health and recovery of weaned piglets post-infection.

**Figure 6:**
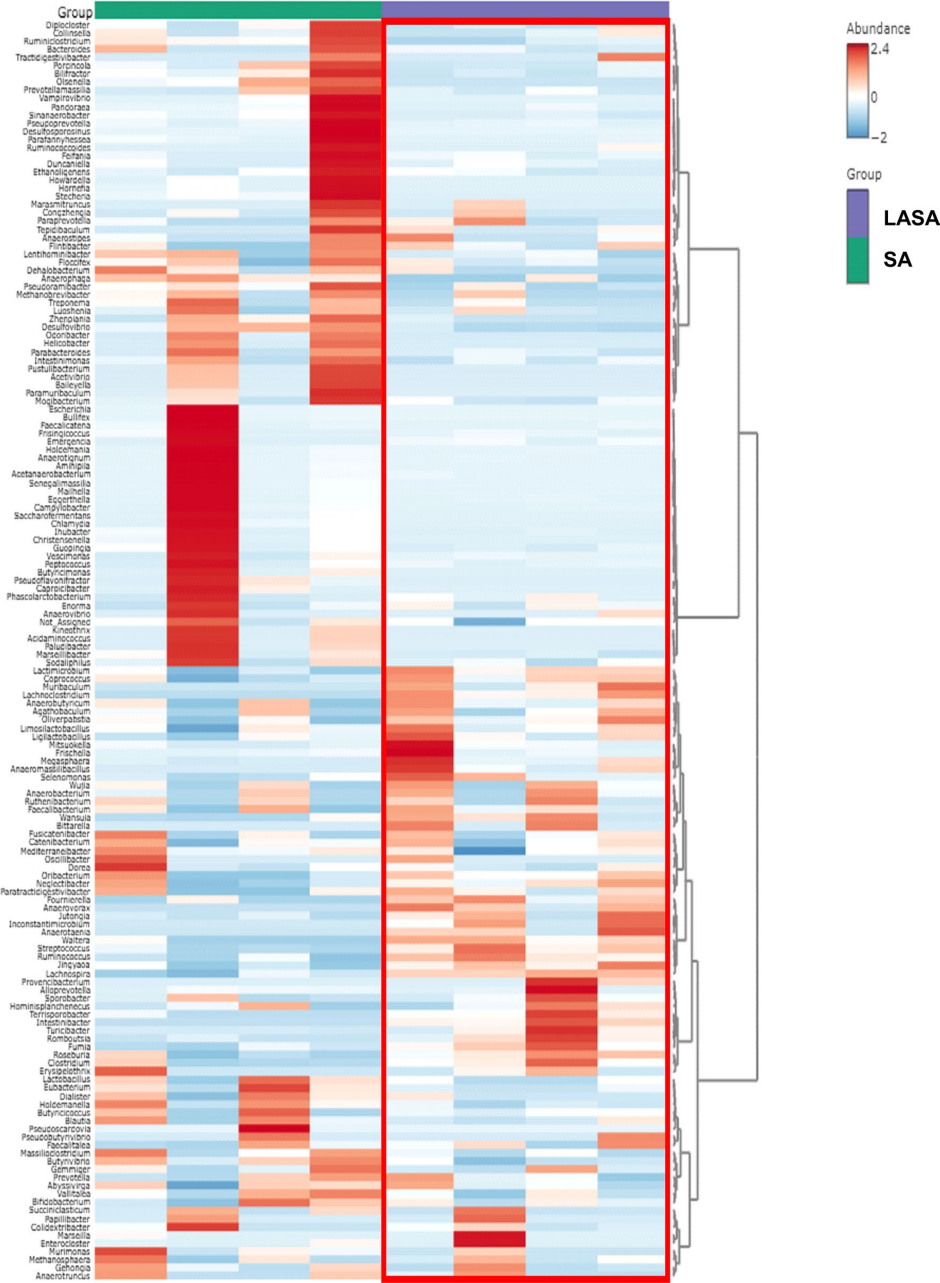
Hierarchical clustering heatmap on day 14 at genus levels using Ward and Euclidean parameters. The red color represents high abundance and blue color represents low abundance. The vertical axis represents the taxonomy level, and the horizontal axis represents the aggregated individuals according to the treatment. The red rectangle represents the unique cluster that showed the higher relative abundances of taxa in the LASA group compared to those in the SA group.

**Figure 7:**
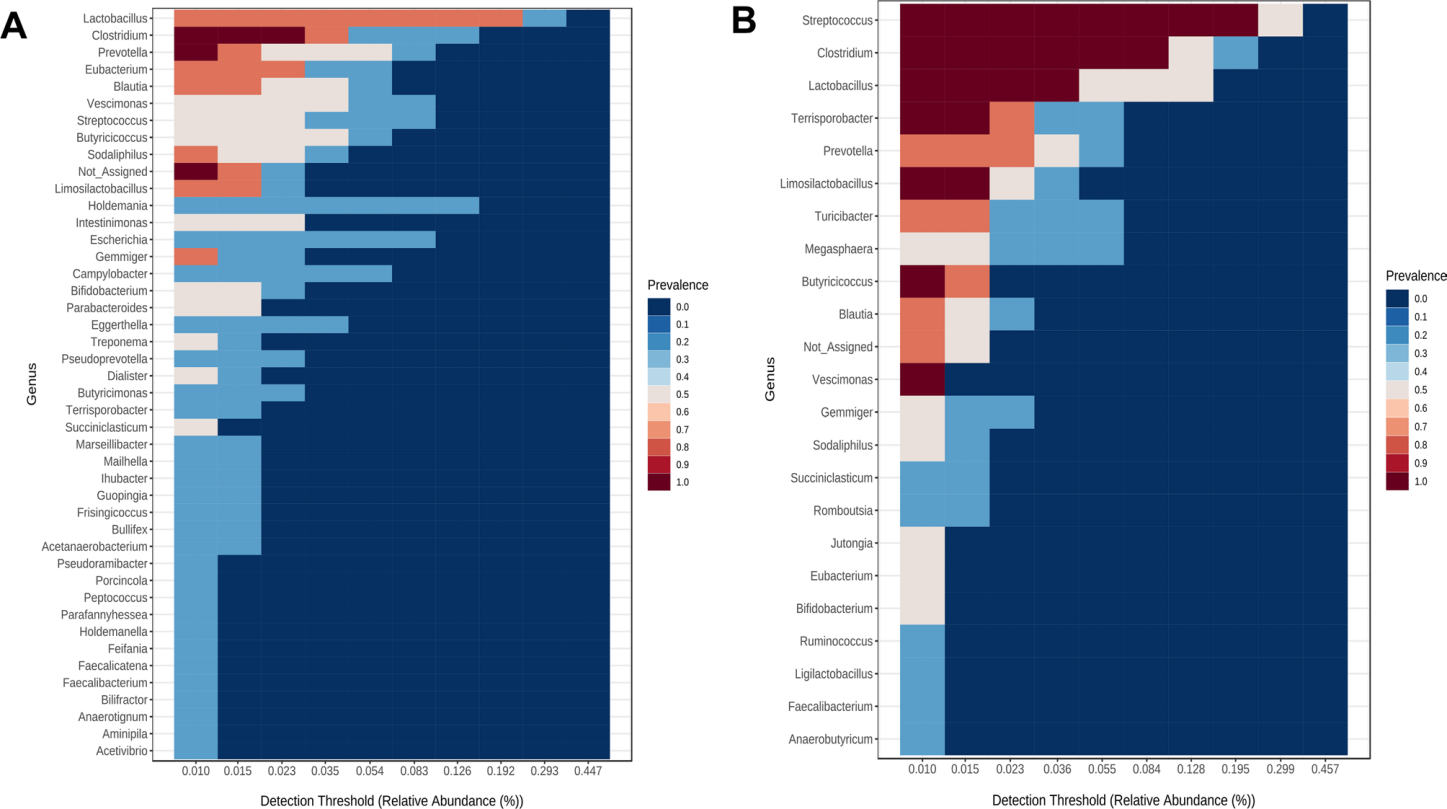
Core microbiome in different groups of weaned piglets. Heatmap depicting the core ASVs and their prevalence at different detection thresholds in the (**A**) SA, (**B**) LASA groups.

To help visualize the varying abundances of bacterial genera across the treatment groups, a hierarchical clustering heat map was generated (Fig. 6). Analysis of the core microbiome was conducted at the genus level, utilizing sample prevalence and relative abundance thresholds of 20% and 0.02%, respectively. In all experimental groups, five core bacterial genera were identified: *Streptococcus*, *Clostridium*, *Lactobacillus*, *Prevotella*, and *Kineothrix*. However, *Escherichia*, *Campylobacter*, and *Treponema* were found exclusively in the SA group and were not present in the LASA group (Fig. 7A,B). These findings indicate that dietary supplementation with LA significantly modulates the gut microbiota composition in weaned piglets challenged with ST.

### LA supplementation regulates the gut-related metabolic function of weaned piglet

**Figure 8:**
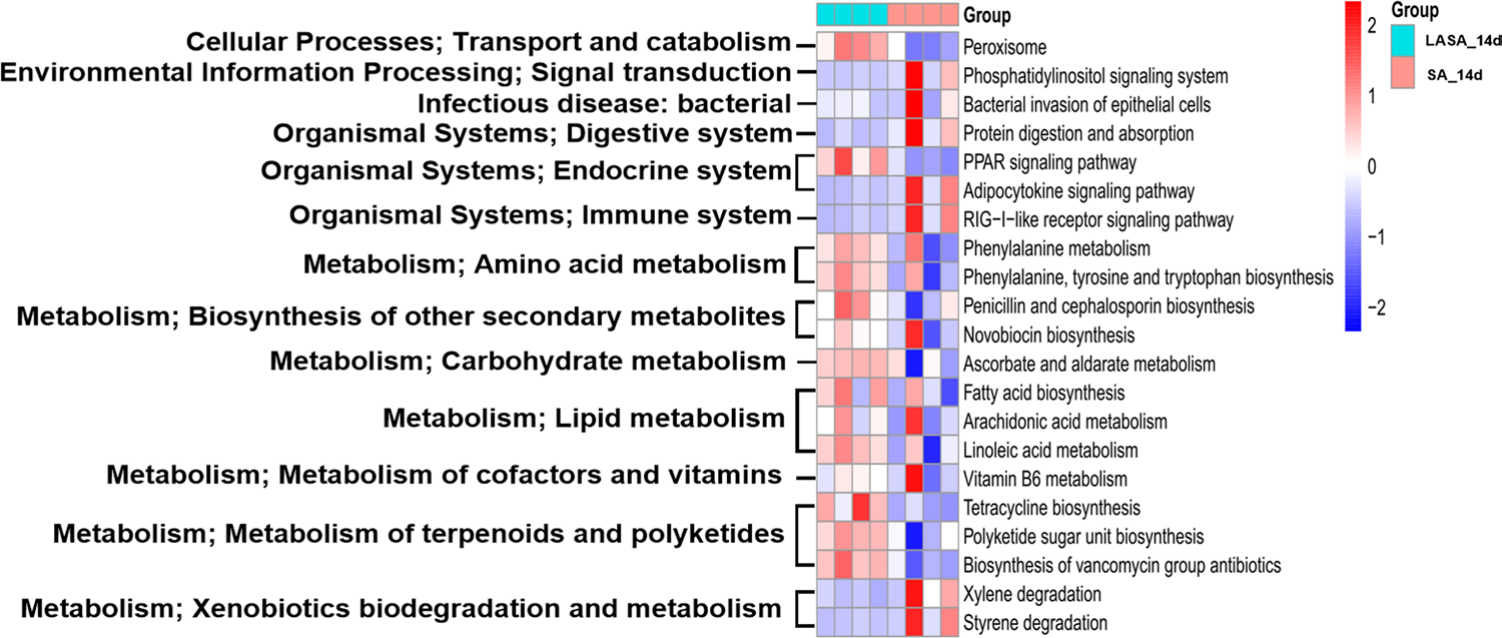
Effect of LASA and SA on gut microbiota that regulates functional pathways in weaned piglets. Based on the analysis of 16S ribosomal RNA V3-V4 gene expression level in the fecal sample, the PICRUSt2 software was used to predict the functional pathway. Heat maps of different functional pathways were compared between the two groups.

**Figure 9:**
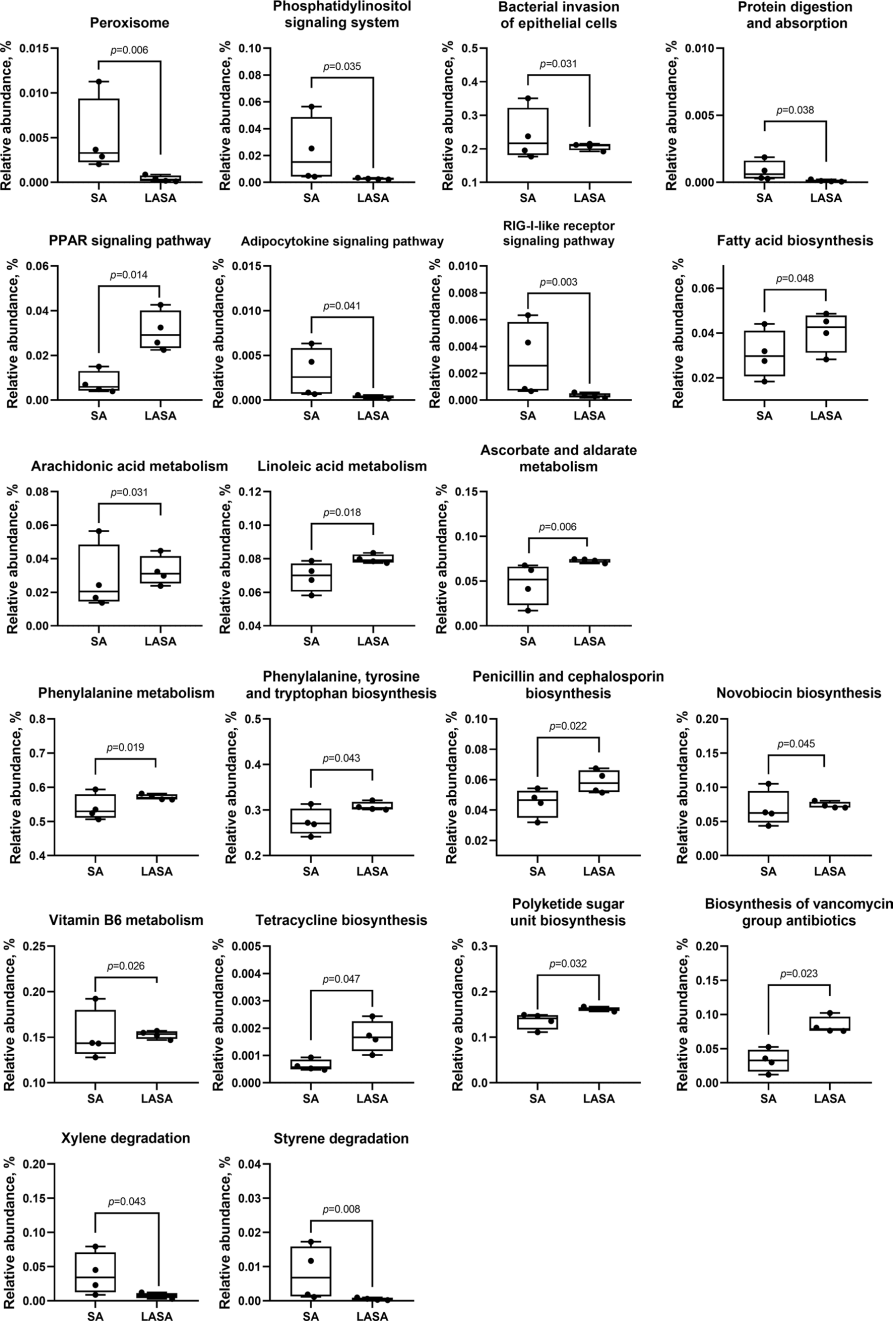
The substantial differences in metabolic functions between the SA and LASA groups includes 21 pathways.

To predict the metabolic function of the differential gut microbiota in weaned piglets supplemented with LA, a functional assessment using PICRUSt2 was performed to analyze the KEGG pathway abundance. The heatmap showed that the pathways in the top 13 categories, including transport and catabolism, signal transduction, immune system, endocrine system, infectious disease, terpenoids, polyketides, cofactors, vitamins, carbohydrates, amino acids, and lipid metabolism, were affected by the intestinal microbiota in each group (Fig. 8). Figure 9 shows 21 subcategories of differential metabolism. Compared to those in the LASA group, factors associated with the metabolism of phosphatidylinositol, adipocytokine, and RIG-1-like receptor signaling pathways and bacterial invasion rates of epithelial cells were upregulated in the group infected with ST. In contrast, LA supplementation significantly attenuated the ETEC-induced upregulation of these pathways (*P* < 0.05). Notably, the LASA group upregulated the metabolism of several pathways, including those of penicillin, cephalosporin, and novobiocin biosynthesis; cofactors and vitamins (vitamin B6); carbohydrates (ascorbate and aldarate); amino acids (phenylalanine, tyrosine, and tryptophan); lipids (fatty acid, arachidonic acid, and linoleic acid); and terpenoids and polyketide (tetracycline and vancomycin) biosynthesis. These results showed that LA supplementation significantly attenuated ST-stimulated upregulation of detrimental metabolic pathways while enhancing the metabolism of antibiotics, vitamins, carbohydrates, and amino acids, suggesting a potential protective and restorative effect on gut microbiota metabolism during ST infection.

## Discussion

Microbiota-derived SCFAs are key element for the maintain intestinal homeostasis and gut health during the intestinal infection^22,28,29^. In our previous study (in vitro), LA showed antimicrobial activity by producing SCFAs (data not published). Therefore, this study aimed to evaluated the potential of LA as a probiotic alternative for swine nutrition, offering protective effects to the gut microbiota against ST infection.

In this study, we assessed the effect of reducing ST infections by administering LA supplementation to weaned piglets. ST infection decreases herd productivity and may reduce economic losses in the pig industry^31^. Our primary focus was on the ADG of weaned pigs infected with ST. LA supplementation significantly improved the ADG of weaned piglets. This result is similar to those of other studies on probiotic feeding^32,33^. Simultaneously, we examined the presence of diarrhea, a prominent indicator of ST gastrointestinal infection^34^. Although more than half of the SA group exhibited diarrhea throughout the experimental period, the LASA group displayed only one or no clinical instances.

While examining the growth performance and disease indicators that proved effective in reducing the symptoms of salmonellosis, we conducted a fecal culture analysis to demonstrate a reduction in ST infection. Throughout the experiment, LA-supplemented weaned piglets (LASA group) consistently exhibited lower levels of fecal ST, and these differences were particularly significant at 2 dpi. The initial peak of fecal shedding in the LASA group occurred between 2 and 5 dpi, whereas the SA group showed shedding from 0 to 2 dpi. The early stage of ST infection in pigs is marked by a rapid acute phase, in which the pathogen is released in comparatively elevated amounts in the feces^8^. Numerous studies have highlighted the potential of probiotics to reduce the number of intestinal ST levels^32,35^. These results suggest that LA supplementation may prevent ST colonization during the feeding period, and subsequently reduce fecal shedding. *Salmonella* counts slightly increased in piglets at 14 dpi. We attribute this increase to potential reinfection from the animal surroundings. Environmental contamination has been identified as a significant factor in *Salmonella* carriage among piglets. Such environmental infection has been shown to be an important factor in *Salmonella* carriage in piglets^34,36^.

In this study, the concentration of fecal SCFA (acetate, propionate, and butyrate) in the LASA group was higher than that in the LA group, although no statistical significance was observed. ST have been reported to both utilize and decrease microbiota-derived butyrate, leading to an alteration in the composition of the gut microbiota. This, in turn, prompts a shift in the intestinal epithelium towards lactate fermentation^37,38^. Furthermore, adequate concentrations of butyrate and propionate have been shown to mitigate ST-induced gut inflammation^37,39^. This suggests that LA supplementation may not sufficiently prevent butyrate consumption by ST, although an increase of SCFA produced by bacteria was observed in our LEfSe results. Nevertheless, LA supplementation could play a role in reducing the severity of symptoms and bacterial colonization in weaned piglets.

The histological scores of the SA and LASA groups showed mild differences in the intestine. Although histopathological changes such as inflammation and villus atrophy were observed in both the SA and LASA groups, ulceration was more severe in the LASA group than in the SA group. The acute phase of *Salmonella* infection is accompanied by an inflammatory response. As the disease progresses, conditions such as ulcerative proctitis may develop^40,41^. These findings suggest that LA supplementation reduces the severity of the infection, although the intestine may not have fully recovered during this period.

To protect weaned piglets from salmonellosis, it is important to preserve a stable structure of the gut microbiome. Several studies have reported that *Salmonella* infections can disrupt microbial structures in piglets^42,43^. In the present study, 16S rRNA gene analysis revealed that LA supplementation did not affect the alpha diversity of swine infected with *Salmonella*. However, PCoA, which is indicative of beta diversity, showed that the intestinal microbial communities of the SA (*Salmonella* infected) and LASA (LA-pre-supplemented and *Salmonella* infected) groups were significantly different (*P* < 0.05). Zhang et al*.*^44^ suggested that pretreatment with *Lactobacillus rhamnosus* GG prevented the disruption of the microbial structure in swine caused by *Salmonella*, which is consistent with our study. Liu et al.^45^ discovered that the administration of *Lactiplantibacillus plantarum* modulated intestinal bacterial populations in mouse models challenged with *Salmonella* infection. These findings suggest that LA supplementation can reduce the severity of salmonellosis in swine by maintaining the gut microbiome stability. Furthermore, taxonomic analysis revealed that *Salmonella* challenge increased the relative abundance of Proteobacteria and decreased that of Bacillota at the phylum level. Typically, an enriched proportion of Proteobacteria, including genera such as *Escherichia*, *Campylobacter*, and *Salmonella*, is associated with intestinal disorders^46–48^. Furthermore, core microbiome analysis showed that *Escherichia* and *Campylobacter* were exclusively detected in the SA group but not in the LASA group. Salmonella-induced intestinal inflammation induced by *Salmonella* may create conditions favorable for the proliferation of Proteobacteria. Nitric oxide, produced during the inflammatory response, is converted to nitrate, which promotes the growth of nitrate reductase gene-having *Escherichia*^49,50^. Furthermore, increased blood flow to the inflamed intestine can lead to oxygen accumulation, favoring facultative anaerobes such as Proteobacteria, thereby disrupting the anaerobic balance and altering the bacterial structure^1^. Our findings suggest that dietary LA supplementation can counteract these effects by reducing Proteobacteria abundance and enhancing Bacillota levels, which along with Bacteroidetes, are key indicators of a balanced intestinal community and overall host health^52,53^. Hierarchical clustering analysis further supported these results, revealing distinct microbial distributions between the SA and LASA groups, implying that LA supplementation could help maintain gut microbiota homeostasis in *Salmonella* infected piglets.

LEfSe analysis was performed to compare the differentially abundant bacteria between the SA and LASA groups. Consistent with the taxonomic analysis results, *Salmonella* infection significantly increased the abundance of Proteobacteria and Bacteroidetes (*P* < 0.05) at the phylum level and the proportions of *Escherichia*, *Campylobacter*, *Desulfovibrio*, and *Treponema* at the genus level. Several studies have indicated that challenge with *Salmonella* increases the relative abundance of Proteobacteria in swine^43,54^. Moreover, fecal–oral pathway is a common route of transmission for *Campylobacter* from sows to piglets, leading to enteritis, particularly in piglets lacking colostrum^55,56^. Riboulet-Bisson et al.^57^ and Zhang et al.^58^ showed that probiotic treatment could inhibit the growth of *Treponema* spp. in weaned piglets, which is consistent with our results. Furthermore, *Desulfovibrio*, an anaerobic bacterium that produces hydrogen sulfide in the gut, may play a role in enhancing the competitive advantage of *Salmonella*. This is due to its contribution to the production of tetrathionate, an electron acceptor that supports the anaerobic respiration of *Salmonella*, as described in the introduction. By producing hydrogen sulfide, which is converted into tetrathionate during inflammation, *Desulfovibrio* inadvertently supports the proliferation of *Salmonella*, which can utilize tetrathionate for respiration, thus outcompeting other microorganisms^59^. Tang et al.^60^ reported that a high proportion of *Desulfovibrio* in the gut could reduce pig productivity. Our study demonstrated that LA administration significantly decreased the abundance of these harmful bacteria. Moreover, the LEfSe analysis indicated that LA supplementation enriched the proportion of major inhabitant (*Streptococcus*, *Clostridium*, *Terrisporobacter*, *Megasphaera* and *Coprococcus*)^61–64^, host-beneficial (*Limosilactobacillus*, *Bifidobacterium* and *Ruminococcus*)^65–67^ and SCFAs producing bacteria (*Jutongia*, *Blautia*, and *Mitsuokella*)^68–70^ in pigs. Our findings suggest that LA supplementation plays a crucial role in alleviating the negative effects of *Salmonella* infection on the gut microbiota of swine by decreasing the abundance of harmful microorganisms and increasing the proportion of beneficial bacteria. This shift in microbial communities could potentially support our results regarding the SCFAs content in feces.

To predict the metabolic function of the differentially abundant gut microbiota between the SA and LASA groups, we analyzed the KEGG gene function using PICRUSt2 based on 16S rDNA gene sequences. Our results revealed that dietary LA supplementation significantly increased the proportion of carbohydrates (ascorbate and aldarate), amino acids (phenylalanine, tyrosine, and tryptophan), cofactors, vitamins (vitamin B6), and rate of lipid (arachidonic acid, linoleic acid, and fatty acid biosynthesis) metabolism, compared to those observed in the SA group. Ascorbate and aldarate metabolisms are crucial carbohydrate metabolic pathways that can alleviate cellular damage caused by oxidative reactions resulting from aerobic metabolism and contamination^71^. Moreover, several studies have reported that amino acids (such as phenylalanine and tyrosine) can be considered potential precursors of SCFAs and can serve as sources for the TCA cycle^72,73^. Moreover, phenylalanine, tyrosine, and tryptophan metabolism is associated with an increased abundance of beneficial bacteria^74,75^ and helps prevent gut inflammation^76,77^. Vitamin B6 is a vital component of cellular metabolism^78^, which contributes to fatty acid biosynthesis^79^. Consistent with these results, linoleic acid plays several roles in lipid metabolism in the gut environment due to its status as an essential fatty acid including cell membrane composition^80^, inflammatory modulation^81^, and energy source of gut epithelial cells^82^. Furthermore, the administration of LA enhances peroxisome metabolism, which preserves the integrity of cells from pathogens^83^, maintains the balance of reactive oxygen species, and protects cells from oxidative stress^84^. Gut bacteria can produce antimicrobial peptide compounds that are considered target-specific. Antimicrobial peptides are classified as ribosomal or non-ribosomal, based on their biosynthesis^85^. In this study, LA treatment was predicted to upregulate the biosynthesis of antimicrobial non-ribosomal peptides (penicillin, cephalosporin, novobiocin, tetracycline, and vancomycin) in piglets. These antimicrobials preferentially inhibit the proliferation of pathogenic bacteria such as Proteobacteria and Spirochetes. Consistent with our results, there was an abundance of bacterial invasion into the epithelial cells (*P* < 0.05). These findings suggest that LA supplementation can potentially modulate the gut microbiome of piglets challenged with *Salmonella*, enhancing nutritional metabolism and beneficial antimicrobial peptide production.

Our study demonstrates that *L. argentoratensis* AGMB00912 is an effective probiotic for reducing ST infection in weaned piglets. It significantly improved growth performance and gut health, underscoring its potential as an alternative to antibiotics. These findings suggest that LA significantly improved growth performance and gut health of ST-challenged piglets, underscoring its potential as an alternative to antibiotics. However, this was a pilot study with a limited sample size and only one method of feeding LA was conducted. Therefore, further large-scale studies and various supplementation methods are required to determine the most effective way to use LA in the pig industry and to confirm its beneficial effects under non-challenged commercial conditions.

## Methods

### Animal experiment

Eight 25-day-old castrated male piglets (Landrace × Yorkshire, 5.86 ± 1.07 kg) were purchased from the same herd of one commercial farm. Before starting the experiment, the pigs were moved to facility, and fecal and blood samples were collected. All weaned pigs were confirmed to be negative for *Salmonella*. The facilities were overseen by designated veterinarians, ensuring a consistent 12-h light–dark cycle and maintaining a stable relative humidity (55% ± 10%) and temperature (21 °C ± 2 °C). The facility consists of separate rooms, each size at 4 m^2^ where equipment and feed separately to prevent contamination. Workers change clothes and take a shower before entering each room. After 3 days, all 28-day-old piglets were randomly divided into two groups and separated in the isolated room (LASA, n = 4; SA, n = 4): Both groups were fed a normal diet throughout the experimental period. The LASA group received an additionally feeding of 1.0 × 10^8^ colony forming units (CFU) of *L*. *argentoratensis* AGMB00912 via a oral gavage (Hauptner AG, Langenthal, Switzerland) once daily, starting from the first day of the experiment and continuing for the consecutive days. The concentration was determined using hemocytometer and diluted with PBS. Weaned piglets in both the SA and LASA groups were orally inoculated using same method described above, with 1 × 10^9^ CFU of ST strain LT2 (ATCC 19,585) at 10-day post feeding. Body weight gain and clinical signs were monitored from 0 to 14 days post-ST inoculation (dpi). Subsequently, all ST-infected piglets were euthanized using T-61 (MSD, N.Y., USA), in accordance with the recommended protocols to ensure humane endpoints. Euthanasia was followed by immediate necropsy to collect tissue samples at 14 days post-infection (dpi). All the animal experiments were approved by the Animal Ethics Committee of the National Institute of Animal Science, Republic of Korea (approval no. NIAS 2021-503). In addition, all methods were performed in accordance with the relevant guidelines and regulations, ensuring compliance with ethical standards for animal research. All experiments were performed in alignment with named guidelines, regulations, and specifically adhered to the ARRIVE guidelines.

### Sampling collection and evaluation of *Salmonella* shedding

Fecal samples for monitoring the shedding of *Salmonella* were collected on days 0, 2, 5, 8, and 14 dpi. Additionally, on day 14, after euthanizing the piglets, intestinal tissues (jejunum, ileum, cecum, and colon) and fresh fecal samples for metagenomic analysis were collected and stored at − 80 °C until analysis. Thereafter, 1 g of fecal matter was inoculated into Rappaport–Vassiliadis broth (RV; BD, Sparks, MD, USA) and incubated at 42 °C for 48 h. The one loop (10 ul) of the RV culture was streaked onto CHROMagar Salmonella Plus (CHROMagar, Paris, France). DNA was extracted from the mauve colonies grown on the agar plate, and the colonies were identified as ST using the AccuPower Salmonella spp. 3-Plex PCR Kit (Bioneer, Daejeon, Korea).

### Histopathology

Jejunum, ileum, cecum, and colon tissue samples collected from necropsied piglets at 14 dpi were fixed in 10% neutral-buffered formalin and embedded in paraffin wax. The sections were 4-μm-thick and stained with hematoxylin and eosin using a standard laboratory protocol. Each intestinal segment was evaluated for five histopathological parameters: severity of inflammation, severity of ulceration, presence of villus shortening, submucosal inflammation and crypt abscess, as described by Argüello H et al.^57^ with modifications (Supplementary Table 1). The total score of the intestine was obtained by adding each score of the intestine segments (jejunum to colon).

### Measurement of fecal SCFA concentration

The concentrations of three types of SCFAs (acetate, propionate, and butyrate) were measured using an Agilent 6890 B gas chromatograph (Agilent Technologies, Santa Clara, CA, USA). Methanol was added to the fecal samples at a 1:10 ratio and homogenized at 250 rpm for 3 h. After homogenate was spined down, the supernatant was collected and filtered through 0.45-μm PTFE syringe filter. Then, 5 μL of the solution was injected into a 30 m × 0.32 mm × 0.5-μm DB-FFAP capillary column (Agilent Technologies, USA). The injector and detector were operated at 220 °C and 250 °C with 5:1 split ratio, respectively. Nitrogen was used as the carrier gas at a flow rate of 1.0 mL/min. The column temperature was maintained at 100 °C for 10 min at initial phase, followed by increased as 10 °C/min to 250 °C for 3 min.

### 16s rRNA gene sequencing analysis

Fecal DNA was extracted using a DNeasy PowerSoil Kit (Qiagen, Hilden, Germany) according to the manufacturer’s instructions. Amplicons of the V3–V4 hypervariable region were generated and sequenced using the Illumina 16S metagenomic sequencing library preparation protocol, which includes the primer sequences 5′-CCTACGGGNGGCWGCAG-3′ for the forward primer and 5′-GACTACHVGGGTATCTAATCC-3′ for the reverse primer. Paired-end sequencing of the amplicons was performed using the MiSeq platform (Illumina, San Diego, CA, USA).

### Bioinformatic analysis

Post sequencing on the Illumina MiSeq platform, indices were used to demultiplex samples, generating paired-end FASTQ files for each sample. Using Cutadapt (v3.2), sequencing adapters and primer sequences targeting the forward and reverse regions of the gene were removed. The forward and reverse reads were trimmed to 250 bp and 200 bp respectively. To correct sequencing errors, the DADA2 (v1.18.0) package in R (v4.0.3) was employed, discarding reads with expected errors greater than 2. Finally, corrected paired-end sequences were merged, chimeric sequences were identified and removed using DADA2’s consensus method, and ASVs were generated. The ASVs were analyzed using BLAST + (v2.9.0) with the National Center for Biotechnology Information 16 s rRNA database used to assign taxonomic information based on the highest similarity with the subjects in the database. The taxonomy assignment was discarded if the best hit from the database showed a query coverage or identity of less than 85%. For multiple alignments of amplicon sequence variant sequences, the MAFFT (v7.475) program was used, and a phylogenetic tree was generated using the FastTreeMP (v2.1.10) program. Using the abundance and taxonomic information of the amplicon sequence variants, various microbial community comparative analyses were conducted utilizing QIIME2 (v1.9). To ascertain species diversity and evenness levels within the microbial communities of the samples, the Shannon and Inverse Simpson indices were calculated. The alpha diversity was verified using rarefaction curves and Chao1 values. Using weighted and unweighted UniFrac distances as a basis, beta diversity was quantified within the comparison groups to estimate microbial community variations among the samples. Visualization of the interrelations between samples was achieved using principal coordinate analysis (PCoA) and the unweighted pair group method with the arithmetic mean tree. To identify specific biomarkers distinguishing between SA and LASA groups, linear discriminant analysis effect size (LEfSe) was performed, using the microbiomeMarker (v1.2.1) R package. LDA scores that ranked differential taxa from the phylum to the genus level were projected onto a cladogram, visualized with GraPhlAn (v1.1.3, Python v2.7), and the differential species level was displayed as a histogram (Prism, v8.0.1). Metabolic functional pathways were predicted using the phylogenetic investigation of communities by reconstruction of unobserved states (PICRUSt2) pipeline (v2.5.2). The assignment of metabolic functional pathways adhered to the Kyoto Encyclopedia of Genes and Genomes (KEGG) Ortholog (KO) database, and the KEGG abundance data for all predicted pathways were recalibrated to relative abundance (%).

### Statistical analysis

Significant changes in the average daily gain (ADG), histopathological lesion scores, SCFA concentrations, and ST fecal shedding log number between the LASA and SA groups were compared using Student's t-test using SPSS software (version 26.0; IBM, Armonk, NY, USA). The group effect on gut microbiota diversity was evaluated using Kruskal-Wallist test. Beta diversity significance was determined using the analysis of similarities based on both unweighted and weighted UniFrac distances. All tests were considered statistically significant at *P*-values of < 0.05. The LEfSe analysis employed an LDA effect size cut-off of ≥ 3 and an alpha of 0.05 for the initial Kruskal–Wallis sum-rank test, followed by the Wilcoxon rank-sum test. Core microbiota analysis was performed using default parameters with 20% sample prevalence and 0.2% relative abundance. Differences in the predicted metagenomic functions between the groups were analyzed using the R software package ALDEx2, with significance set at *P*-values of < 0.05. Given the pilot nature of this study, the limited sample size of piglets was primarily driven by budget constraints and the exploratory aims of the research. Despite the small sample size, this study was intended to provide trends that could inform larger, statistically powered studies.

### Supplementary Information

Supplementary Figures.

Supplementary Tables.

Supplementary Legends.

## Data Availability

The high-throughput sequencing data generated in this study have been deposited in the SRA database under the accession number PRJNA1073052.
