# Supplementary figures and images for: Lactiplantibacillus argentoratensis AGMB00912 alleviates salmonellosis and modulates gut microbiota in weaned piglets: a pilot study
Source: Sci Rep. 2024 Jul 5;14:15466. doi: 10.1038/s41598-024-66092-z (PMC11224356; doi:10.1038/s41598-024-66092-z)

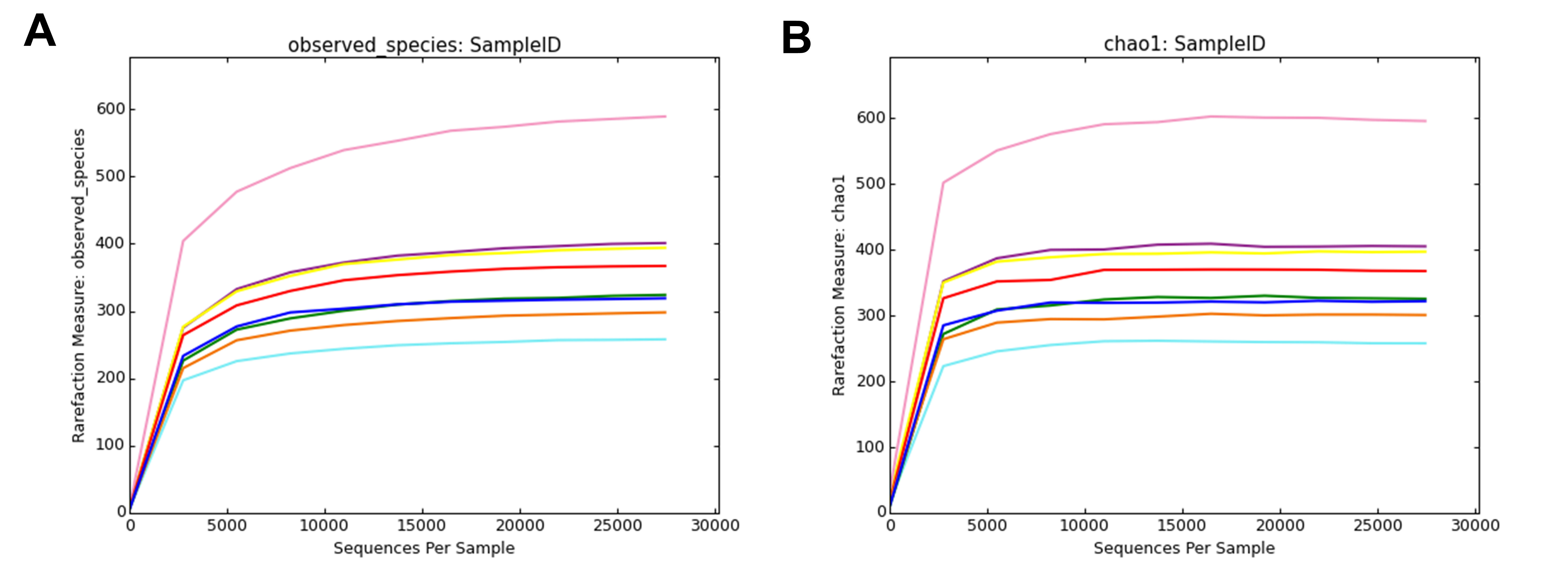

Supplement: Supplementary file 1 — Supplementary Figures. [file 41598_2024_66092_MOESM1_ESM.tif]
