# Supplementary material for: Lactiplantibacillus argentoratensis AGMB00912 alleviates salmonellosis and modulates gut microbiota in weaned piglets: a pilot study
Source: Sci Rep. 2024 Jul 5;14:15466. doi: 10.1038/s41598-024-66092-z (PMC11224356; doi:10.1038/s41598-024-66092-z)
Supplement: Supplementary file 2 — Supplementary Tables. [file 41598_2024_66092_MOESM2_ESM.docx]

**Additional file 2.** Histopathological scoring criteria.

|  | Inflammation | Ulceration |
| --- | --- | --- |
| Criteria | Number of neutrophils  (average at 5 points along the intestine, x 400 magnification) | Number of ulcerations in the crypt  (average at 6 points along the crypt, x10 magnification |
| Score |  |  |
| 0 | 0 | 0 |
| 1 | 1~10 | 1~2 |
| 2 | 11~100 | 3~4 |
| 3 | 100~ | 4~6 |

Additional point: present of villus atrophy (+1), submucosal inflammation (+1), crypt abscess (+1) in the intestine respectively.
