# Supplementary material for: Lactiplantibacillus argentoratensis AGMB00912 alleviates salmonellosis and modulates gut microbiota in weaned piglets: a pilot study
Source: Sci Rep. 2024 Jul 5;14:15466. doi: 10.1038/s41598-024-66092-z (PMC11224356; doi:10.1038/s41598-024-66092-z)
Supplement: Supplementary file 3 — Supplementary Legends. [file 41598_2024_66092_MOESM3_ESM.docx]

Figure S1. Comparative rarefaction analysis of microbial diversity in SA and LASA groups. The number of sequences were normalized to the minimum number of sequences between the SA and LASA groups to account for differences in sampling depth. Each curve represents an individual stool sample. The X-axis represents the number of sequences and Y-axis represents species.
